# Buffalo Academy of Medicine

**Published:** 1894-11

**Authors:** E. J. Gilray

**Affiliations:** Secretary


### BUFFALO ACADEMY OF MEDICINE.

### SECTION ON SURGERY.

Reported by E. J. GILRAY, M. D., Secretary.

THE regular monthly meeting of the surgical section of the Academy of Medicine was held in the Academy Parlors, October 2, 1894, Dr. M. HARTWIG in the chair.

Present: Drs. Hartwig, Hopkins, Sherman, Dowd, Shroetter, Hayd, Grosvenor, Van Peyma, Goldberg, Potter, Carroll, Snow, Mulford, Paterson, Carter, Eugene Smith, Le Van, Renner, Tremaine, Krauss, Chauncey Smith, Dwyer and Gilray.

*Papers.*—The Value of Extension and Counter-Extension in the Treatment of Fractures of the Humerus, Dr. J. W. Grosvenor. Volkmann's Splint in Fracture of the Thigh, Dr. H. E. Hayd. Ambulatory Treatment in Fractures of the Lower Extremities, Dr. Marcell Hartwig.

DISCUSSION.

Dr. TREMAINE: Did not, as a rule, approve of any special splint or design, but the surgeon should have sufficient ingenuity to improvise to meet exigencies of case. Took exception to Dr. Hayd's wholesale denunciation of plaster of Paris casts. Every fracture must be treated on its own merits.

Dr. EUGENE SMITH: Where certain complications existed, plaster of Paris casts were the only ones that could be used in fracture of the thigh.

Dr. HAYD: Seemed to him that there was no place for a plaster of Paris cast, except in a maniac or irresponsible person. No matter how carefully it be put up, the surgeon has no direct proof that bones are in apposition.

Dr. HARTWIG cautioned the younger members against being seduced by Dr. Hayd's scathing disapproval of the plaster of Paris cast. The danger in cast is in not making it high enough.

Dr. HAYD presented a specimen of extra-uterine pregnancy.

Meeting adjourned at 10.30 P. M.

